# Chitosan-GSNO Nanoparticles and Silicon Priming Enhance the Germination and Seedling Growth of Soybean (*Glycine max* L.)

**DOI:** 10.3390/plants13101290

**Published:** 2024-05-07

**Authors:** Senabulya Steven, Mohammad Shafiqul Islam, Amit Ghimire, Nusrat Jahan Methela, Eun-Hae Kwon, Byung-Wook Yun, In-Jung Lee, Seong-Hoon Kim, Yoonha Kim

**Affiliations:** 1 Department of Food Security and Agricultural Development, Kyungpook National University, Daegu 41566, Republic of Korea; stevensenabulya@gmail.com; 2 Department of Applied Biosciences, Kyungpook National University, Daegu 41566, Republic of Korea; shafik.hort@gmail.com (M.S.I.); ghimireamit2009@gmail.com (A.G.); methela.ag@nstu.edu.bd (N.J.M.); eunhaekwon@naver.com (E.-H.K.); bwyun@knu.ac.kr (B.-W.Y.); ijlee@knu.ac.kr (I.-J.L.); 3 Department of Integrative Biology, Kyungpook National University, Daegu 41566, Republic of Korea; 4 National Agrobiodiversity Center, National Institute of Agricultural Sciences, RDA, Jeonju 5487, Republic of Korea; shkim0819@korea.kr; 5 Upland Field Machinery Research Center, Kyungpook National University, Daegu 41566, Republic of Korea

**Keywords:** seed priming, germination, seedling growth, gene expression, GA, ABA

## Abstract

Soybean, a major legume crop, has seen a decline in its production owing to challenges in seed germination and the development of seedlings. Thus, in this study, we systematically investigated the influence of various chitosan–S-nitrosoglutathione (chitosan-GSNO) nanoparticle (0, 25, 50, and 100 µM) and Si (0, 0.5, and 1 mM) priming concentrations on soybean seed germination and seedling growth over five different priming durations (range: 1–5 h at each concentration). Significant differences were observed in all parameters, except seedling diameter, with both treatments. Seed germination was significantly enhanced after 3 h of priming in both treatments. The final germination percentage (FGP), peak germination percentage (PGP), vigor index (VI), seedling biomass (SB), hypocotyl length (HL), and radical length (RL) of 100 μM chitosan-GSNO-nanoparticle-primed seeds increased by 20.3%, 41.3%, 78.9%, 25.2%, 15.7%, and 65.9%, respectively, compared with those of the control; however, the mean germination time (MGT) decreased by 18.43%. Si priming at 0.5 mM increased the FGP, PGP, VI, SB, HL, and RL by 13.9%, 55.17%, 39.2%, 6.5%, 22.5%, and 25.1%, respectively, but reduced the MGT by 12.29% compared with the control treatment. Chitosan-GSNO and Si treatment up-regulated the relative expression of gibberellic acid (GA)-related genes (*GmGA3ox3* and *GmGA2ox1*) and down-regulated that of abscisic acid (ABA)-related genes (*GmABA2*, *GmAAO3*, and *GmNCED5*). Chitosan-GSNO and Si application increased bioactive GA_4_ levels and simultaneously reduced ABA content. Hence, the use of exogenous chitosan-GSNO nanoparticles and Si as priming agents had a beneficial effect on seed germination and seedling growth because of the up-regulation in the expression of GA and down-regulation in the expression of ABA. Additional research is needed to understand the combined impact of Si and chitosan-GSNO nanoparticles, including their effects on the expression levels of other hormones and genes even in the later growth stage of the crop.

## 1. Introduction

Soybean (*Glycine max* L. Merr.) is a commercially significant food and oilseed crop, and it contains approximately 20–22% oil; 40–45% protein; and substantial amounts of carbohydrates (30–33%) and fiber (6–8%) [1,2,3]. It plays a vital role in upholding food security, alleviating poverty, replenishing soil nutrients, and fostering national economic development [4,5].

Seeds are the primary means of transferring advanced genetic material for crop production. Therefore, rapid and synchronous seed germination is crucial for agricultural output and seedling emergence [6]. The germination process entails reciprocal interactions between the embryo and endosperm, whereby the endosperm functions as a sensor of the environment, regulating the embryo’s growth, while the embryo governs the degradation of the endosperm [7]. However, seed germination or emergence remains a crucial aspect for seedling growth and crop yield [8]. Seed priming potentially enhances this aspect, and it involves the regulation of water content and seed temperature to control germination. Pre-planting seed treatment enables the regulated absorption of moisture by seeds, facilitating the initial phase of germination while preventing root emergence through the seed coat [9,10]. Seed priming has extensively been studied, and its benefits have been well documented [11]. It has been shown to enhance germination under challenging environmental conditions, reduce thermo- and photo-dormancy, and improve the seed’s ability to compete with weeds and pathogens [11,12]. The value of seed priming is reported to be strongly associated with plant physiology, genotype, and vigor [13]. Notably, the length of time required for seed priming varies with plant species, as the priming procedure is customized to suit the specific needs of each crop [14]. Therefore, novel approaches with properties that promote plant growth need to be developed. This study focused on seed priming with Si and chitosan-based S-nitrosoglutathione (GSNO) nanoparticles as a plausible means of improving use efficacy and enhancing plant growth [15,16].

Si, the second-most abundant element on the Earth’s surface, has the prospects to intensify vegetative growth, development, and resistance against abiotic and biotic stresses [17,18]. Furthermore, Si utilization offers additional advantages, such as improved photosynthesis, delayed senescence, and a reduced transpiration rate [19]. Moreover, Si application has emerged as a relatively novel strategy that exhibits promise in alleviating significant limitations to crop production across different varieties [20]. The application of an optimal concentration of Si reportedly improves soybean seed germination and seedling growth [14,21]. The use of Si has been found to have a positive impact on the germination of seeds and the subsequent growth of seedlings in *Medicago sativa* L. [22], maize [23], barley [24], and lentil [25]. Chitosan biopolymer is a novel plant elicitor or bio-stimulant that is gaining recognition owing to its non-toxic and biodegradable nature [16]. Chitosan is a polysaccharide produced from deacetylated chitin from various sources, including crustaceans, insects, and fungi. Chitosan nanoparticles have been reported to enhance seed germination and minimize stress responses, such as peroxidase activity, phytoalexins, and chitinases [26,27]. The utilization of chitosan nanoparticles has been documented to enhance the process of seed germination and promote the seedling growth of wheat [27], rice [28], maize [29], and broad bean [30]. Studies on the response of soybean seed to priming with chitosan nanoparticles during germination and seedling growth are limited. From the above discussion, we have grasped the importance of chitosan nanoparticles and Si application. Hence, knowing the importance of chitosan nanoparticles and Si in seed germination and seedling growth parameters, in this research, our main objective was to find the best priming duration and concentration among Si and chitosan-GSNO nanoparticles along with their role in GA and ABA contents and their hormone-related gene expression. Furthermore, to our knowledge, this is the first research to look at the effect of chitosan-GSNO nanoparticles as a priming agent for seed germination. Therefore, this preliminary research underscores the necessity for future studies to investigate the combined effects of optimal priming agents not only during seed treatment, but also during subsequent growth stages, particularly under plant stress conditions.

## 2. Results

### 2.1. Analysis of Variance (ANOVA) of Germination and Seedling Growth

ANOVA revealed significant differences in the final germination percentage (FGP), peak germination percentage (PGP), mean germination time (MGT), vigor index (VI), seedling biomass (SB), root length (RL), and hypocotyl length (HL) (*p* < 0.05–0.001) following priming with chitosan-GSNO nanoparticles and Si at various priming durations and concentrations (Table 1). No significant differences in seedling diameter (SD) were observed in both treatments. Similarly, across all parameters, except SB, no significant interaction effect was found between priming concentration and duration after chitosan-GSNO nanoparticle and Si treatment (*p* < 0.01). Based on the ANOVA results, we concluded that priming with chitosan-GSNO nanoparticles and Si at different concentrations and durations plays a significant role in soybean seed germination and seedling growth.

### 2.2. Effects of Chitosan-GSNO Nanoparticles and Si on the FGP

**Figure 1:**
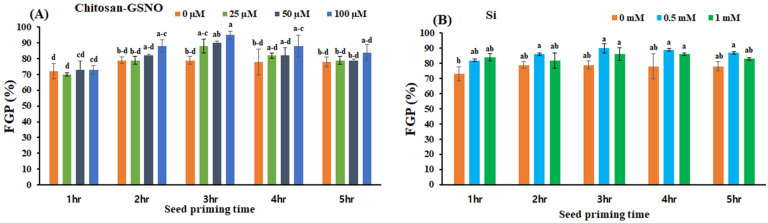
Effects of chitosan-GSNO nanoparticles and Si on the final germination percentage (FGP) of soybean. (**A**) FGP (%) for chitosan-GSNO. (**B**) FGP (%) for Si. Different lowercase letters indicate significant differences at *p* ≤ 0.05 based on Duncan’s multiple-range test, whereas similar lowercase letters represent insignificant differences. Error bars indicate standard errors; 0 mM/μM indicates the control treatment.

Chitosan-GSNO nanoparticle- and Si-treated seeds exhibited significantly increased FGPs at all priming durations and concentrations compared with the controls (Figure 1). Chitosan-GSNO nanoparticles increased the FGP by 20.3%, 12.8%, and 7.7% when administered for 3, 4, and 5 h at 100 μM, respectively, compared with the control treatment (Figure 1A). The highest FGP (95%) was obtained when seeds were primed for 3 h with 100 μM chitosan-GSNO nanoparticles, followed by FGPs of 90%, 88%, and 79% with 50 μM, 25 μM, and the control treatment, respectively. Similarly, the FGP increased by 13.9%, 14.1%, and 11.5% in seeds primed with 0.5 mM Si for 3, 4, and 5 h, respectively, compared with that in the control (Figure 1B). The maximum FGP (90%) occurred in seeds primed with 0.5 mM Si for 3 h compared with an FGP of 79% for the control. Among all priming durations, 3 h of seed priming displayed the most significant effect on the FGP in both chitosan-GSNO-nanoparticle- and Si-treated soybean seeds.

### 2.3. Influence of Chitosan-GSNO Nanoparticles and Si on the PGP and MGT

**Figure 2:**
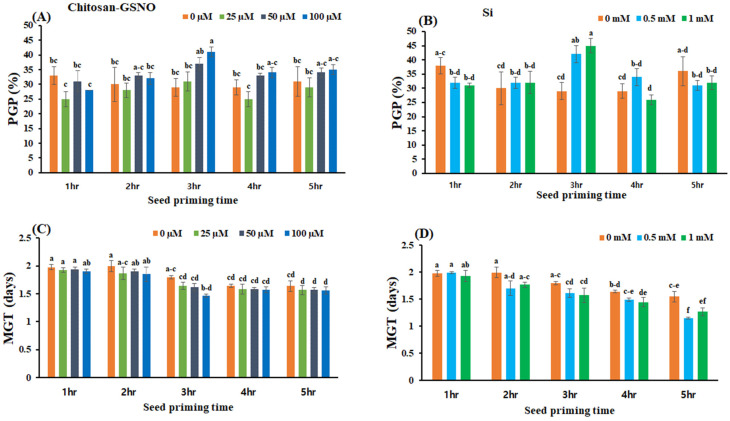
Effects of chitosan-GSNO nanoparticles and Si on the peak germination percentage (PGP) and mean germination time (MGT). (**A**) PGP (%) for chitosan-GSNO; (**B**) PGP (%) for Si; (**C**) MGT (days) for chitosan-GSNO; (**D**) MGT (days) for Si. Different lowercase letters indicate significant differences at *p* ≤ 0.05 based on Duncan’s multiple-range test, whereas similar lowercase letters represent insignificant differences. Error bars indicate standard errors; 0 mM/μM indicates the control treatment.

Chitosan-GSNO-nanoparticle- and Si-treated seeds showed significant variations in the PGP and MGT across all priming durations and concentrations compared with the controls (Figure 2). At 100 μM, chitosan-GSNO nanoparticles increased the PGP by 41.3%, 17.2%, and 12.9% when administered for 3, 4, and 5 h, respectively, compared with the control treatment. At 0.5 mM, Si increased the PGP by 55.17%, 17.3%, and 6.6% when administered for 3, 4, and 2 h, respectively, compared with the control treatment (Figure 2A,B). The highest PGP (41%) was attained when seeds were primed for 3 h with 100 μM chitosan-GSNO nanoparticles, followed by PGPs of 37%, 31%, and 29% with 50 μM, 25 μM, and the control treatment, respectively. Likewise, the maximum PGP (45%) was obtained in seeds primed with 0.5 mM Si for 3 h compared with 29% in the control seeds. The shortest MGT (1.46 days) was achieved after chitosan-GSNO treatment at 100 μM for 3 h, followed by MGTs of 1.62, 1.64, and 1.79 days with 50 μM, 25 μM, and the control treatment, respectively. Furthermore, with Si, the shortest MGT (1.59 days) was obtained after priming at 0.5 mM for 3 h, followed by MGTs of 1.62 and 1.79 days with 1.0 mM and the control treatment, respectively. Chitosan-GSNO nanoparticle treatment at 100 μM shortened the MGT by 18.43%, 4.87%, and 5.48% after priming for 3, 4, and 5 h, respectively, compared with the control treatment. Si treatment at 0.5 mM shortened the MGT by 12.29% and 11.28% after priming for 3 and 2 h, respectively, compared with the control treatment; considerably lower MGT-reduction rates were observed after 1, 4, and 5 h of priming at all concentrations (Figure 2C,D).

### 2.4. Effects of Chitosan-GSNO Nanoparticles and Si on the VI and SB

**Figure 3:**
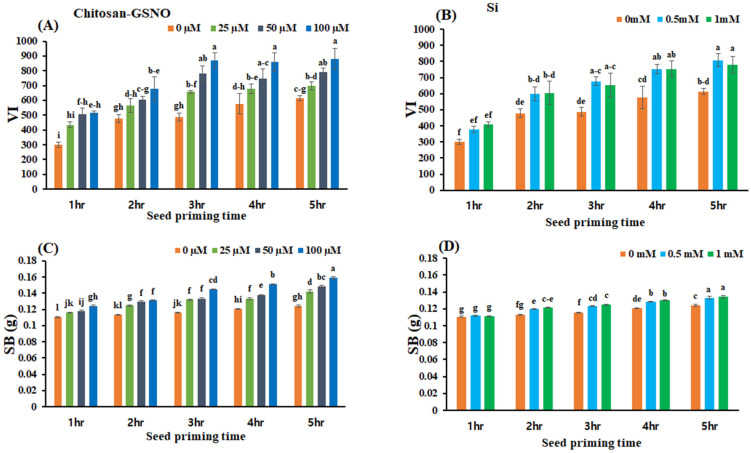
Effects of chitosan-GSNO nanoparticles and Si on the vigor index (VI) and seedling biomass (SB). (**A**) VI for chitosan-GSNO; (**B**) VI for Si; (**C**) SB (g) for chitosan-GSNO; (**D**) SB (g) for Si. Different lowercase letters indicate significant differences at *p* ≤ 0.05 based on Duncan’s multiple-range test, and similar lowercase letters represent insignificant differences. Error bars indicate standard errors; 0 mM/μM indicates the control treatment.

Chitosan-GSNO nanoparticle- and Si-primed seeds presented significantly elevated VI and SB values at all priming durations (range: 1–5 h) and concentrations (Figure 3). After priming with 100 μM chitosan-GSNO nanoparticles for 3, 4, and 5 h, the VI significantly increased by 78.9%, 48.9%, and 43.2%, respectively, compared with that after control treatment; the highest VI value (879.46) was achieved after priming with 100 μM chitosan-GSNO nanoparticles for 5 h (Figure 3A). Priming with 0.5 mM Si for 3, 4, and 5 h substantially increased the VI by 39.2%, 30.2%, and 31.4%, respectively, compared with the control treatment. The highest VI value (807.25) was achieved after priming with 0.5 mM Si for 5 h (Figure 3B). SB significantly increased with increasing priming concentration and duration. At 100 μM, priming with chitosan-GSNO nanoparticles for 3, 4, and 5 h increased the SB by 25.2%, 25.3%, and 28.0%, respectively, compared with the control treatment. Likewise, at 0.5 mM, Si priming for 3, 4, and 5 h elevated the SB by 6.5%, 6.6%, and 7.2%, respectively, compared with the control treatment (Figure 3C,D).

### 2.5. Impact of Chitosan-GSNO Nanoparticles and Si on HL, RL, and SD

**Figure 4:**
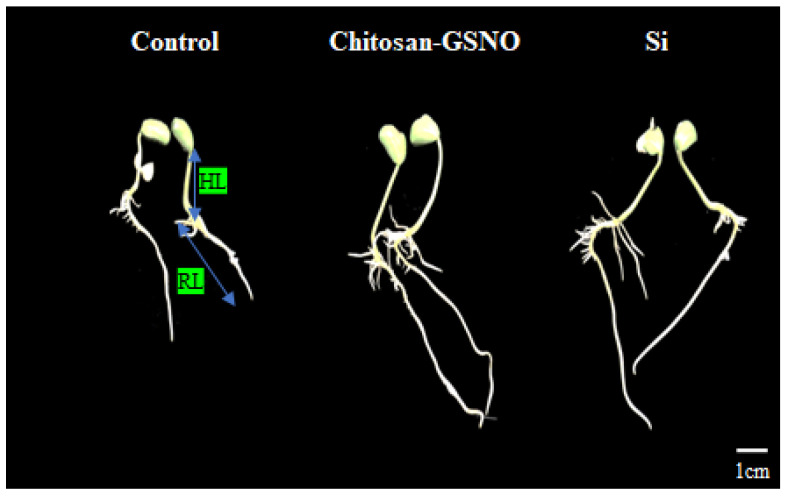
Effects of chitosan-GSNO nanoparticles and Si on hypocotyl length (HL) and root length (RL) in soybean seedlings after 5 days (120 h). The scale bar is given on the right bottom corner.

**Figure 5:**
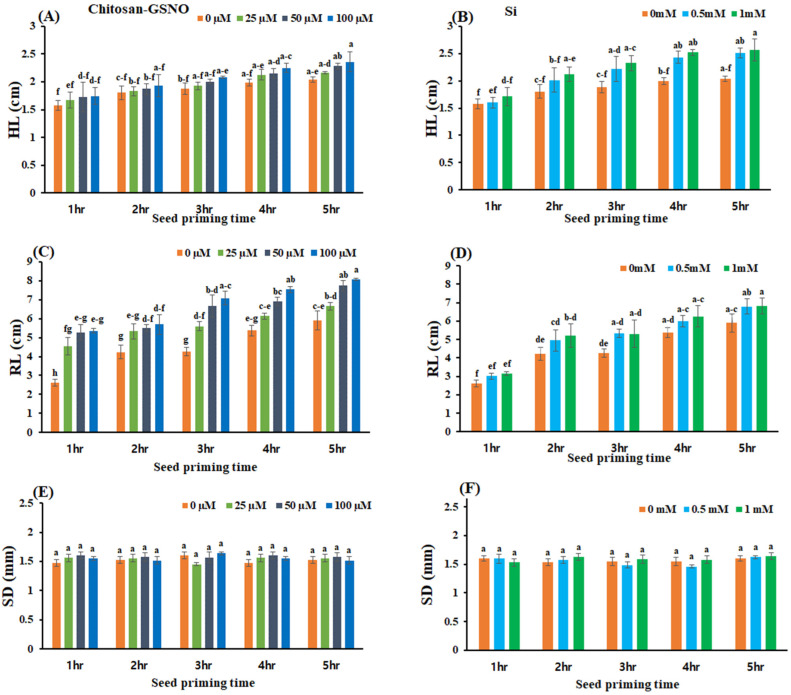
Effect of chitosan-GSNO nanoparticles and Si on hypocotyl length (HL), root length (RL), and seedling diameter (SD). (**A**) HL for chitosan-GSNO; (**B**) HL for Si; (**C**) RL for chitosan-GSNO; (**D**) RL for Si; (**E**) SD for chitosan-GSNO; (**F**) SD for Si. Different lowercase letters indicate significant differences at *p* ≤ 0.05 based on Duncan’s multiple-range test, while similar lowercase letters represent insignificant differences. Error bars indicate standard errors; 0 mM/μM indicates the control treatment.

Chitosan-GSNO-nanoparticle- and Si-primed seeds yielded statistically significant differences in HL and RL across all priming durations and concentrations (Figure 4 and Figure 5). Following the 5-day treatment, variations in the HL and RL were detected after chitosan-GSNO nanoparticle and Si priming compared with that after control treatment (Figure 4). At 100 μM, chitosan-GSNO nanoparticle priming for 3, 4, and 5 h increased the HL by 10.6%, 13.1%, and 15.7%, respectively, compared with the control treatment. Similarly, the HL in seeds treated with 0.5 mM Si for 3, 4, and 5 h increased by 17.6%, 22.0%, and 22.5%, respectively, compared with that in the control (Figure 5A,B). The maximum HL (2.4 cm) was recorded after priming with 100 μM chitosan-GSNO nanoparticles for 5 h, while the minimum HL (1.6 cm) was observed after control treatment for 1 h. With Si treatment, the longest HL (2.6 cm) was recorded after priming at a concentration of 1 mM for 5 h, while the shortest HL (1.6 cm) was observed after control treatment for 1 h. The RL increased by 65.9%, 40.2%, and 36.8% after priming with 100 μM chitosan-GSNO for 3, 4, and 5 h, respectively, compared with that after control treatment. Correspondingly, the RL increased by 25.1%, 11.9%, and 14.9% after treatment with 0.5 mM Si for 3, 4, and 5 h, respectively, compared with that after control treatment (Figure 5C,D). The longest RL (8.1 cm) was observed after priming with 100 μM chitosan-GSNO for 5 h, while the shortest RL (2.6 cm) was identified after control treatment for 1 h. In the case of Si-treated seeds, the maximum RL (6.8 cm) was observed after priming at 1 mM for 5 h, while the minimum RL (2.6 cm) was identified after control treatment for 1 h. No significant difference in the SD was observed after applying chitosan-GSNO nanoparticles or Si at all priming durations and concentrations (Figure 5E,F).

### 2.6. GA- and ABA-Pathway-Related Gene Expression in Soybean Seeds

**Figure 6:**
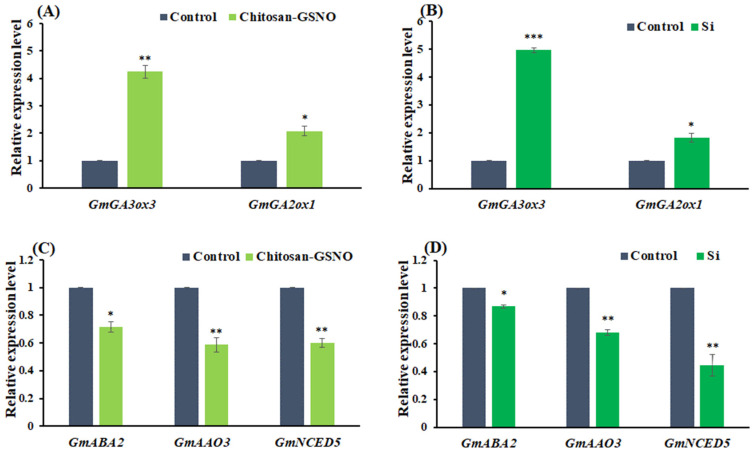
Relative gene expression levels of GA (*GmGA3ox3*, *GmGA2ox1*)- and ABA (*GmABA2*, *GmAAO3*, *GmNCED5*)-related genes in soybean seeds primed with chitosan-GSNO nanoparticles and Si compared with those in the control. (**A**,**B**) Relative expression levels of *GmGA3ox3* and *GmGA2ox1* in chitosan-GSNO- and Si-primed seeds compared with those in the control; (**C**,**D**) relative expression levels of *GmABA2*, *GmAAO3*, and *GmNCED5* in chitosan-GSNO- and Si-primed seeds compared with those in the control. Student’s *t*-test was used for statistical analysis. * Difference is significant at the 0.05 level; ** difference is significant at the 0.01 level, *** difference is significant at the 0.001 level.

Previous studies have established the pivotal roles of gibberellic acid (GA) and abscisic acid (ABA) in seed germination [31]. Therefore, we delved deeper into the matter to address the current knowledge gaps regarding the association of the positive influence of chitosan-GSNO and Si on soybean seed germination with the GA and ABA pathways. Consequently, we analyzed the expression levels of GA- and ABA-related genes that influence soybean seed germination. Gene expression was observed after 3 h of priming of soybean seeds. Quantitative real-time polymerase chain reaction (qRT-PCR) revealed a significant improvement in the relative expression levels of GA biosynthesis genes, namely *GmGA3ox3* and *GmGA2ox1*, after priming with chitosan-GSNO nanoparticles and Si. The expression of these genes was up-regulated in primed seeds compared with that in the control group (Figure 6A,B). *GmGA3ox3* and *GmGA2ox1* expression levels increased 4.2- and 2.1-fold with chitosan-GSNO nanoparticle priming and 4.9- and 1.8-fold with Si treatment, respectively. Furthermore, we evaluated the relative expression levels of key genes involved in ABA biosynthesis. Briefly, qRT-PCR revealed that chitosan-GSNO nanoparticle and Si priming down-regulated the relative expression levels of ABA biosynthesis genes, namely *GmAAO3*, *GmABA2*, and *GmNCED5*, compared with the control treatment (Figure 6C,D). Chitosan-GSNO nanoparticle treatment suppressed *GmABA2*, *GmAAO3*, and *GmNCED5* gene expression by 29%, 42%, and 41%, respectively, compared with the control treatment. Likewise, Si treatment down-regulated *GmABA2*, *GmAAO3*, *and GmNCED5* gene expression by 14%, 32%, and 55%, respectively, compared with the control treatment. Through careful examination, chitosan-GSNO and Si have been found to exert an inhibitory effect on ABA biosynthesis during soybean seed germination. Collectively, the results indicate that the introduction of exogenous chitosan-GSNO nanoparticles and the implementation of Si priming positively impact GA biosynthesis and concurrently suppress ABA biogenesis.

### 2.7. Impact of Chitosan-GSNO Nanoparticle and Si Priming on Endogenous Bioactive GA_4_ and ABA

**Figure 7:**
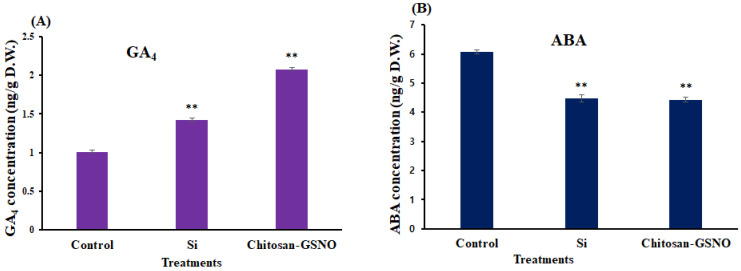
Endogenous bioactive GA_4_ and ABA contents of soybean seeds primed with chitosan-GSNO nanoparticles and Si compared with those of the control. Content of (**A**) endogenous GA_4_ and (**B**) ABA in primed seeds compared with that in the control. Student’s *t*-test was used for statistical analysis. ** Difference is significant at the 0.01 level.

The endogenous active GA_4_ and ABA contents of the primed soybean seeds were quantified. The endogenous bioactive GA_4_ content of primed seeds was significantly increased by chitosan-GSNO nanoparticle (100 μM) and Si (0.5 mM) priming compared with that of the control (Figure 7A). The results revealed that chitosan-GSNO nanoparticles and Si increased the active GA_4_ content of primed seeds by 100.7% and 42%, respectively, compared with the control. Moreover, the bioactive ABA content of primed seeds was significantly reduced by chitosan-GSNO nanoparticle and Si treatment compared with that of the control (Figure 7). Therefore, chitosan-GSNO nanoparticles (100 μM) and Si (0.5 mM) reduced the active ABA content of primed seeds by 26.9% and 26.2%, respectively, compared with the control (Figure 7B).

## 3. Discussion

Seed priming is one of the beneficial and effective hydration techniques used to stimulate seed germination. Crop seeds trigger various physiological responses involved in controlling hydration and drying during priming; thus, priming can initiate pre-germination metabolic processes for early germination [32]. Therefore, seed-priming techniques have multiple benefits pertaining to water reduction and cost. According to various studies, priming potentially exerts beneficial effects on field crops such as wheat [33], maize [34], mung bean [35], barley [36], lentils [37], and cucumbers [38]. However, several factors, such as priming duration, type of priming chemical, and concentration, among others, significantly influence priming effectiveness for seed germination [11,12]. Therefore, we sought to establish the optimal concentration and duration of chitosan-GSNO and Si priming for enhancing soybean seed germination and promoting early seedling growth.

Currently, chitosan biopolymer is an emerging non-toxic bio-stimulant in plants; hence, it has been broadly used in several studies. Methela et al. [16] formulated chitosan-GSNO nanoparticles. GSNO is known as a potential nitric oxide (NO) donor, and NO maintains various physiological responses, such as resistance to drought, salinity, and heavy metal stress [16,39,40,41]. However, NO is a considerably unstable signal molecule; thus, NO donors (e.g., sodium nitroprusside and GSNO) function in plants as stress scavengers [39]. Therefore, our experiment also used chitosan-GSNO to elucidate seed-priming effects. According to our findings, the application of chitosan-GSNO nanoparticles caused significant increases in the FGP, PGP, SB, VI, RL, and HL and a decrease in the MGT. Notably, the use of a NO donor, that is chitosan-based GSNO at a concentration of 100 µM, demonstrated substantial increases in the FGP, PGP, VI, SB, HL, and RL and a significant decrease in the MGT. Previous studies have yielded similar results in (1) the seed germination percentage, vitality index, shoot length, and root length of wheat after the application of chitosan nanoparticles [27]; (2) the seed germination percentage and vigor index of maize [42]; and (3) the vigor index of pearl millet [43]. Additionally, chitosan nanoparticles potentially stimulate seed germination in *Zea mays*, *Brassica rapa*, and *Pisum sativum* [44]. Nevertheless, to date, the literature documenting the effects of chitosan-based GSNO nanoparticles on seed germination remains drastically limited, except for a study by [16], where these nanoparticles were found to alleviate drought tolerance in soybean seedlings. Si is one of the most useful seed-priming agents that improve seed germination and seedling parameters. According to our results, seed priming with 0.5 mM Si increased the FGP, PGP, SB, VI, RL, and HL and reduced the MGT. Parallel results were obtained with the application of exogenous Si, which promoted the (1) seed germination, VI, RL, and shoot height in maize [45]; (2) seed germination and VI in cucumber [15]; (3) seed germination in lentil [46]; and (4) plant height and biomass in soybean [47]. In addition, Si application reportedly enhances seedling growth (i.e., plant height, biomass, tiller number, and photosynthesis) in barley [24]. The probable reasons for the enhanced germination features of soybean may be related to cell division, water imbibition, DNA synthesis, and the activity of different enzymes [48,49,50,51].

In most cases of seed germination, the plant hormones GA and ABA play crucial roles [31]. Endogenous GA is synthesized in three different plant cell organelles, namely the plastid, endoplasmic reticulum, and cytosol [52]. In that pathway, numerous enzymes catalyze precursors and are regulated by several genes, such as *GA2ox*, *GA20ox*, *GA3ox*, and *GA13ox* [53]. Among them, *GA3ox* and *GA2ox* regulate extremely important responses. *GA3ox* catalyzes non-bioactive GA (GA_9_ and GA_20_) to bioactive GA (GA_4_ and GA_1_), while *GA2ox* participates in the degradation of bioactive GA to non-bioactive GA (GA_34_ and GA_8_) [54,55]. Therefore, both genes act as key regulators of various physiological responses, such as cell growth, cell division, stem elongation, and seed germination, among others. [56,57]. ABA is also a considerably important plant hormone that induces seed dormancy, leaf abscission, and stomata regulation, among others [58]. Zeaxanthin is a precursor of ABA, and it is converted to xanthoxin in the plastid with the help of several genes, such as *ABA4* and *NCED*s; thereafter, xanthoxin migrates to the cytosol [59]. *ABA2* and *AAO3* subsequently regulate the conversion of xanthoxin to abscisic aldehyde and ABA, respectively. To elucidate the molecular and physiological responses to seed priming with chitosan-GSNO nanoparticles and Si, we examined the relative gene expression of GA- and ABA-pathway-related genes and evaluated the GA_4_ and ABA contents of soybean. According to our results, GA- and ABA-biosynthesis-related genes yielded contrasting results. GA-biosynthesis-related genes (*GmGA3ox3* and *GmGA2ox1*) exhibited significant up-regulation in primed seeds compared with those in the control; however, ABA-biosynthesis-related genes (*GmABA2*, *GmAAO3*, and *GmNCED5*) displayed significant down-regulation compared with those in the control. Consequently, the up- and down-regulation of GA- and ABA-biosynthesis-related genes increased endogenous GA_4_ contents and decreased that of ABA in chitosan-GSNO-nanoparticle- and Si-primed seeds compared with that in the control. Chitosan nanoparticles have been documented to positively regulate GA content and negatively regulate ABA content during seed germination [60], as well as promote GA expression in *Phaseolus vulgaris* seed germination [61]. Similarly, Si application has also been documented to improve seed germination and seedling growth by regulating GA and ABA in cucumber [15] and *Glycyrrhiza uralensis* [62]. Upon comprehensively considering all findings, GSNO nanoparticles and Si can be presumed to enhance soybean seed germination and seedling growth by regulating GA and ABA concentrations in soybean seedlings via the up- and down-regulation of the respective hormones’ biosynthesis-related genes.

## 4. Materials and Methods

### 4.1. Plant Materials, Treatments, and Experimental Design

The seeds of soybean cultivar Pungsanamul were used in this study. The study was conducted in the crop production laboratory of Kyungpook National University, Republic of Korea, in June–July 2023 using the top-of-paper germination method [63]. Chitosan-GSNO nanoparticles and Si in the form of sodium metasilicate pentahydrate (Na_2_O_3_Si.5H_2_O; Sigma-Aldrich, Zwijndrecht, The Netherland) were used as priming agents. Four concentrations (0, 25, 50, and 100 µM) of chitosan-GSNO nanoparticles and three of Si (0, 0.5, and 1 mM) were used in the experiment, and a concentration of 0 mM/µM represented the control for both treatments. The detailed preparation of chitosan-GSNO nanoparticles has been described in our recently published article [16]. The solutions were homogeneously dispersed in distilled water for 15 min using a vortex shaker [64]. The experiment followed a completely randomized design, with four replications.

### 4.2. Seed Priming, Germination Testing, Growth Conditions, and Data Collection

**Figure 8:**
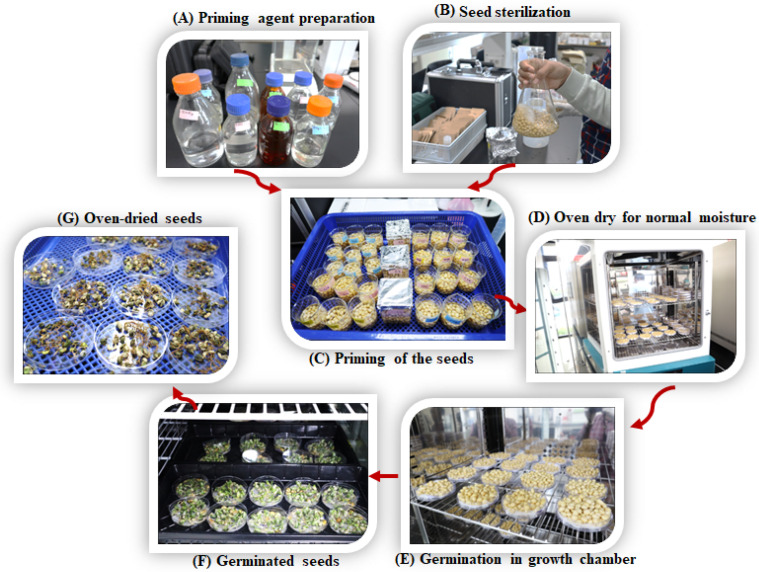
Soybean seed priming and germination. (**A**) Priming agent preparation, (**B**) seed sterilization, (**C**) seed priming, (**D**) oven drying at 25 °C to achieve normal moisture, (**E**) seed germination in growth chamber, (**F**) germinated seeds, and (**G**) oven-dried seedlings.

The seeds were primarily surface-sterilized with 0.5% sodium hypochlorite (*v*/*v*) for 10 min and then thoroughly washed with distilled water [65]. Subsequently, the seeds were soaked in chitosan-GSNO nanoparticle (0, 25, 50, and 100 µM) and Si (0, 0.5, and 1 mM) solutions for 1–5 h. A seed weight-to-solution volume ratio of 1:5 (*w*/*v*) was maintained [66]. The soaked seeds were drained from the solutions and air-dried back to their near-original moisture content of 10.1% using a forced convection oven (JSON-150, Natural Convection Oven, Gongju-City, Korea) at 25 °C over a 48 h period. Twenty-five primed seeds for each treatment were subjected to germination testing. The seeds were uniformly spread on a 9 cm petri dish on the surface of two-layered tissue paper (substrate) spaced at a distance of at least three-times that of the seed [67]. Thereafter, 4 mL of distilled water was added to the substrate for wetting. The petri dishes were tightly covered and placed in a growth chamber, where the following conditions were maintained: a temperature of 23 ± 2 °C, relative humidity of 60–70%, and photoperiod of 12 light/12 dark hours, as suggested by [68]. The substrate’s moisture level was monitored daily by watering throughout the experiment. Germination counts were recorded at 12 h intervals for up to 120 h from sowing. Seed germination was defined as the emergence of approximately 0.2 cm of the radical through the seed coat [69]. At 120 h (5 days), 10 seedlings were randomly selected from each experimental unit, and different parameters were measured. The overall procedure from seed priming to seed germination is illustrated in Figure 8A–E.

### 4.3. Determination of Germination Parameters

The FGP was measured 120 h after seed sowing by counting normally germinated seedlings and calculating their percentage [70]. The MGT was the average time required for the maximum germination of a seed lot [71]. The PGP was used to measure the highest seed germination percentage obtained at a particular time interval [72].

(1) $$FGP=\frac{N_{g}}{N_{t}}\times 100$$

where *N_g_* is the number of germinated seeds and *N_t_* is the total number of seeds.

(2) $$MGT=\frac{\sum_{i=1}^{k}N_{i}T_{i}}{\sum_{i=1}^{k}N_{i}}$$

where *T_i_* is the time from experiment initiation to the *i*th interval, *N_i_* is the number of germinated seeds at the *i*th interval (the number corresponding to the *i*th interval, but not the accumulated number), and *k* is the total number of time intervals.

(3) $$PGP=\frac{N_{max}}{N_{t}}\times 100$$

where $N_{max}$ is the maximum number of germinated seeds per interval and $N_{t}$ is the total number of seeds.

### 4.4. Determination of Morphological Parameters

Various physiological parameters were measured in this study. The RL (cm) was measured from the hypocotyl–radicle junction to the root cap using a millimetric ruler, and the HL (cm) was measured from the hypocotyl–radicle junction to the cotyledon using a millimetric ruler. The SB (g) was measured by weighing seedling samples previously dried in a forced convection oven (70 °C, 72 h; JSON-150) using a high-precision scale [73]. The SD was measured using digital Vernier calipers to obtain the circumference of the shoot 1 cm above the hypocotyl–radicle junction. The seedling’s VI was used to evaluate the seed’s potential performance during germination and seedling development [74].

(4) VI=FGP×SB

### 4.5. qRT-PCR Analysis of Gene Expression

qRT-PCR was used to analyze the relative gene expression levels of *GmGA3ox3*, *GmGA2ox1*, *GmABA2*, *GmAAO3*, and *GmNCED5* using the primers listed in Table 2. Seed samples were subjected to 3 h of priming with 100 µM chitosan-GSNO nanoparticles and 0.5 mM Si and subsequently ground in liquid nitrogen; thereafter, ribonucleic acid (RNA) was extracted using the TRIzol^®^ reagent (Invitrogen, Waltham, MA, USA). Complementary deoxyribonucleic acid (cDNA) was synthesized using a BioFact™ RT-Kit (BioFact, Yuseong-Gu, Daejeon, Korea), according to the manufacturer’s instructions, with 1 μg of RNA as the starting material. Subsequently, two-step PCR was conducted on an Eco™ real-time PCR machine (BIO-RAD, CFX Duet, Real-Time PCR system, Singapore) using the 2× Real-Time PCR Master Mix containing SYBR^®^ Green l (Solg^TM^, Yuseong-Gu, Daejeon, Korea) with initial and subsequent 40-cycle denaturation steps at 95 °C for 15 min and 20 s, respectively, as well as simultaneous primer annealing and extension at 58 °C for 40 s. To establish a basis for comparison, the actin gene (*GmActin7*, Gene ID: *GLYMA_06G150100*) was used as a reference gene.

### 4.6. Quantification of GA_4_ in Soybean Seeds

Endogenous bioactive GA_4_ was extracted and quantified using a well-established protocol [75]. Treated seed samples were promptly frozen in liquid nitrogen and stored in a −70 °C ultra-low refrigerator (Sanyo-Ultra Low, Moriguchi, Osaka, Japan). After lyophilization, the seed samples were finely ground into a powder. An 8 g lyophilized sample was subsequently extracted with 100% acetone solution (*v*/*v*) for GA_4_ analysis. For analysis, a gas chromatograph (GC) (Hewlett-Packard 6890, 5973N Mass Selective Detector, Santa Clara, CA, USA) with an HA-1 capillary column (30 m × 0.25 mm i.d., 0.25 μm film thickness) was employed. The GC was programmed to initiate at 60 °C for 1 min, followed by ramping at 15 °C/min up to 200 °C and subsequently 5 °C/min up to 285 °C. A helium carrier gas head pressure of 30 kPa was maintained throughout. The GC was directly coupled with a mass-selective detector with an interface, and the source temperatures was set at 280 °C. An ionizing voltage of 70 eV and dwell time of 100 ms were applied. Initially, full-scan mode was used, followed by the monitoring of three major ions of the supplemented hydrogen isotope (Deuterium) [^2^H_2_]. GA internal standards along with endogenous gibberellins were used in subsequent trials. The standard GA_4_ utilized in this study was procured from Professor Lewis N. Mander at the Australian National University in Canberra, Australia. Endogenous GA_4_ content was determined by calculating the peak area ratio of 284/286, and the data are expressed in nanograms/gram (ng/g) dry weight. To ensure precision and reliability, the analysis was conducted thrice with different samples each time.

### 4.7. Quantification of ABA in Soybean Seeds

The endogenous free ABA content of soybean seeds was measured according to the protocol established by Qi et al. [76]. Initially, 1.6 g of lyophilized ground samples was extracted using 30 mL of a solution comprising 95% isopropanol, 5% glacial acetic acid, and 20 ng of ABA. The resulting filtrate was concentrated using a rotary evaporator. The concentrated substance was dissolved in 4 mL of 1 N NaOH solution and washed three times with 3 mL of methylene chloride to remove any lipophilic compounds. The pH of the aqueous phase was adjusted to approximately 3.5 using 6 N HCl, and the resulting solution was subjected to three partitioning steps with EtOAc. The EtOAc extracts were combined and evaporated, and the dried residue was dissolved in phosphate buffer (pH 8.0) before passing through a polyvinylpoly-pyrrolidone (PVPP) column. Subsequently, the phosphate buffer was acidified to pH 3.5 with 6 N HCl and subjected to three more partitioning steps with EtOAc. The resulting extracts were combined, evaporated, and dissolved in phosphate buffer (pH 8.0) before being passed through another PVPP column. After adjusting the pH to 3.5 with 6 N HCl, the phosphate buffer was subjected to three more partitioning steps with EtOAc. The combined EtOAc extracts were evaporated, and the dried residue was dissolved in dichloromethane and filtered through a silica cartridge (Sep-Pak; Water Associates, Milford, MA, USA). Prior to filtration, the cartridge was washed with diethyl ether/methanol (3:2, *v*/*v*) and dichloromethane. ABA was eluted from the cartridge using diethyl ether and methanol (3:2, *v*/*v*). Subsequently, the samples were methylated with diazomethane for GC/MS–SIM analysis using Agilent Technologies’ 6890N network GC system and 5973 network mass selective detector, with quantification performed using ThermoQuset’s (GC 2000 series) lab-based software. The software was instrumental in monitoring responses to ions with mass-to-charge ratios of 162 and 190 for Me-ABA, and 166/194 for Me-[^2^H_6_]-ABA. The analysis was conducted three times, with different samples used in each trial, and the data are expressed in ng/g of dry weight.

### 4.8. Statistical Analysis

The data are presented as the mean and standard error, and graphs were constructed in Microsoft *Excel* (2019). Statistical analysis was performed via two-way ANOVA using the *R* software (version 4.3.1). Significantly different means were determined via Duncan’s multiple-range test using *R-studio*. Statistical significance was set at *p* ≤ 0.05.

## 5. Conclusions

Our study revealed that priming with exogenous chitosan-GSNO nanoparticles and Si enhances seed germination and seedling growth and shortens the germination time of soybean seeds. These treatments stimulate GA biosynthesis and suppress ABA biosynthesis in soybean. Therefore, our findings suggest that both seed priming methods not only enhance seed germination, but also improve early seedling growth, rendering them potentially beneficial for soybean productivity.

## Figures and Tables

**Table 1:** ANOVA of the effects of chitosan-GSNO and Si priming on soybean germination and seedling traits.

**Chitosan-GSNO**									
**Source**	**Df**	**FGP**	**PGP**	**MGT**	**VI**	**SB**	**RL**	**HL**	**SD**
Time	4	6.515	2.528	26.292	24.228	226.5	30.31	9.673	0.27
		<0.001 ***	0.0464 *	<0.001 ***	<0.001 ***	<0.001 ***	<0.001 ***	<0.001 ***	0.76 ^ns^
Conc.	3	3.039	2.436	2.686	25.342	305.7	35.265	2.762	0.292
		0.03581 *	0.04502 *	0.05 *	<0.001 ***	<0.001 ***	<0.001 ***	0.0484 *	0.83 ^ns^
Time × Conc.	12	0.32	0.991	0.244	0.507	9	0.756	0.716	0.896
		0.9828 ^ns^	0.4678 ^ns^	0.9949 ^ns^	0.902 ^ns^	<0.001 ***	0.691 ^ns^	0.6381 ^ns^	0.58 ^ns^
**Si**									
**Source**	**Df**	**FGP**	**PGP**	**MGT**	**VI**	**SB**	**RL**	**HL**	**SD**
Time	4	4.375	2.687	22.598	24.893	150.48	23.256	10.552	0.804
		0.0029 **	0.0431 *	<0.001 ***	<0.001 ***	<0.001 ***	<0.001 ***	<0.001 ***	0.53 ^ns^
Conc.	2	7.89	2.316	7.899	14.732	74.58	4.621	8.165	0.496
		0.00115 **	0.0496 *	0.00115 **	<0.001 ***	<0.001 ***	0.015 *	<0.001 ***	0.60 ^ns^
Time × Conc.	8	0.225	2.084	0.733	0.265	3.71	0.503	0.406	0.427
		0.9844 ^ns^	0.0575 ^ns^	0.6616 ^ns^	0.973 ^ns^	<0.002 **	0.8513 ^ns^	0.9111 ^ns^	0.89 ^ns^

Notes: ns, not significant; * significant (*p* ≤ 0.05); ** significant (*p* < 0.01); *** significant (*p* < 0.001). FGP, final germination percentage; PGP, peak germination percentage; MGT, mean germination time; RL, radical length; HL, hypogeal length; VI, vigor index; SB, seedling biomass; SD, seedling diameter; Conc., concentration; ×, interaction.

**Table 2:** PCR primers used for gene expression.

Primers	Sequence (Forward 5′-3′)	Sequence (Reverse 5′-3′)
*GmGA3ox3*	CTCGCATCTCTTCCTTCTTCC	AATCCAACATCAGCCACATCAG
*GmABA2*	CATAGTCAACAATGCTGGAATCTC	ACCTAAGGCACTTGCTACAC
*GmAAO3*	AACTGAAGAAGACACCAACAAG	CTACGCAAGCACCACAAC
*GmGA2ox1*	TGTGAGCTTCTTGATCTGGTG	GGGAGGGTATTGATTGATCCT
*GmNCED5*	TACTTGTGCATAGCGGAACC	GCACAAAAGCCATCACGTAC
*GmActin7*	GCAAGAACTCGAGACTGCAA	CCAGCAGCTTCCATTCCAAT

## Data Availability

Data are contained within the article.
